# Supplementary material for: Opponent regulation of action performance and timing by striatonigral and striatopallidal pathways
Source: eLife. 2020 Apr 23;9:e54831. doi: 10.7554/eLife.54831 (PMC7180055; doi:10.7554/eLife.54831)
Supplement: Transparent reporting form [file elife-54831-transrepform.docx]

***eLife’s* transparent reporting form**

We encourage authors to provide detailed information *within their submission* to facilitate the interpretation and replication of experiments. Authors can upload supporting documentation to indicate the use of appropriate reporting guidelines for health-related research (see [EQUATOR Network](http://www.equator-network.org/%20)), life science research (see the [BioSharing Information Resource](https://biosharing.org/)), or the [ARRIVE guidelines](http://www.plosbiology.org/article/info:doi/10.1371/journal.pbio.1000412) for reporting work involving animal research. Where applicable, authors should refer to any relevant reporting standards documents in this form.

If you have any questions, please consult our Journal Policies and/or contact us: [editorial@elifesciences.org](mailto:editorial@elifesciences.org).

**Sample-size estimation**

- You should state whether an appropriate sample size was computed when the study was being designed
- You should state the statistical method of sample size computation and any required assumptions
- If no explicit power analysis was used, you should describe how you decided what sample (replicate) size (number) to use

Please outline where this information can be found within the submission (e.g., sections or figure legends), or explain why this information doesn’t apply to your submission:

Explicit power analysis was not performed, but numbers (n = 8 for D1-cre and A2A-cre mice and n = 5 for GFP controls) were based on evaluating the number of animals used in other systems-neuroscience studies of the direct and indirect pathways:

Tecuapetla *et al*. 2014, Nature Communications, max n = 10 mice.

Cui *et al.* 2013, Nature, max n = 4 mice.

Kravitz *et al.* 2013, Nature, max n = 16 mice.

Freeze *et al*. 2015, JNeuroscience, max n = 5 mice.

**Replicates**

- You should report how often each experiment was performed
- You should include a definition of biological versus technical replication
- The data obtained should be provided and sufficient information should be provided to indicate the number of independent biological and/or technical replicates
- If you encountered any outliers, you should describe how these were handled
- Criteria for exclusion/inclusion of data should be clearly stated
- High-throughput sequence data should be uploaded before submission, with a private link for reviewers provided (these are available from both GEO and ArrayExpress)

Please outline where this information can be found within the submission (e.g., sections or figure legends), or explain why this information doesn’t apply to your submission:

In this study, an experiment is defined as a session that had a single combination of stimulation frequency and laser stimulation onset time parameters during the interval (e.g. 10 Hz at 1 second before reward). A biological replication is defined as performing the same combination of stimulation parameters on a unique animal. A technical replication is defined as a repetition of the same combination of parameters on the same animal.

No technical replicates were included in the study, although they were performed when a certain condition was performed during poor licking performance. In the case of technical replication, the replicate that contained the most robust anticipatory licking was used. In this way, we could at least know that the animals were engaged in the task, which was relevant because motivational variables could impact the effects of laser stimulation (Figure 7). The experiment with poorer performance was excluded.

The number of biological replicates is stated in each figure legend.

Timing analysis was performed on a subset of mice (n = 4 D1-cre mice and n = 5 A2A-cre mice) because the study had two cohorts of D1-cre and A2A-cre mice. The first cohort was euthanized before timing experiments could be performed.

No outliers were encountered.

**Statistical reporting**

- Statistical analysis methods should be described and justified
- Raw data should be presented in figures whenever informative to do so (typically when N per group is less than 10)
- For each experiment, you should identify the statistical tests used, exact values of N, definitions of center, methods of multiple test correction, and dispersion and precision measures (e.g., mean, median, SD, SEM, confidence intervals; and, for the major substantive results, a measure of effect size (e.g., Pearson's r, Cohen's d)
- Report exact p-values wherever possible alongside the summary statistics and 95% confidence intervals. These should be reported for all key questions and not only when the p-value is less than 0.05.

Please outline where this information can be found within the submission (e.g., sections or figure legends), or explain why this information doesn’t apply to your submission:

Statistical analysis was clearly reported for each figure in both the legend and in the results section, along with the number of experimental replicates in each group. All figures contained error bars that reflected the SEM.

The majority of our statistical analyses were done using ANOVAs, with appropriate Bonferroni post hoc tests when significant interactions were found.

exact p-values were included in the cases when tests did not detect significant differences. Otherwise, the highest standard p-value thresholds were reported.

(For large datasets, or papers with a very large number of statistical tests, you may upload a single table file with tests, Ns, etc., with reference to sections in the manuscript.)

**Group allocation**

- Indicate how samples were allocated into experimental groups (in the case of clinical studies, please specify allocation to treatment method); if randomization was used, please also state if restricted randomization was applied
- Indicate if masking was used during group allocation, data collection and/or data analysis

Please outline where this information can be found within the submission (e.g., sections or figure legends), or explain why this information doesn’t apply to your submission:

Samples were allocated according to Cre-expression in the striatonigral-projecting or striatopallidal-projecting cell populations.

Experimenters were not blind as to experimental group throughout the experiment.

**Additional data files (“source data”)**

- We encourage you to upload relevant additional data files, such as numerical data that are represented as a graph in a figure, or as a summary table
- Where provided, these should be in the most useful format, and they can be uploaded as “Source data” files linked to a main figure or table
- Include model definition files including the full list of parameters used
- Include code used for data analysis (e.g., R, MatLab)
- Avoid stating that data files are “available upon request”

Please indicate the figures or tables for which source data files have been provided:

Both code and data are available upon request.
